# Structural evaluations and temperature dependent photoluminescence characterizations of Eu^3+^-activated SrZrO_3_ hollow spheres for luminescence thermometry applications

**DOI:** 10.1038/srep25787

**Published:** 2016-05-18

**Authors:** Subrata Das, Sudipta Som, Che-Yuan Yang, Sudam Chavhan, Chung-Hsin Lu

**Affiliations:** 1 Department of Chemical Engineering, National Taiwan University, Taipei, Taiwan, ROC

## Abstract

This research is focused on the temperature sensing ability of perovskite SrZrO_3_:Eu^3+^ hollow spheres synthesized via the sol-gel method followed by heating. The Rietveld refinement indicated that the precursors annealed at 1100 °C were crystallized to form orthorhombic SrZrO_3_. SrZrO_3_ particles exhibited non-agglomerated hollow spherical morphology with an average particle size of 300 nm. The UV-excited photoluminescence spectrum of SrZrO_3_:Eu^3+^ consisted of two regions. One region was associated with SrZrO_3_ trap emission, and the other one was related to the emission of Eu^3+^ ions. The intensity ratio of the emission of Eu^3+^ ions to the host emission (FIR) and the emission lifetime of Eu^3+^ ions were measured in the temperature range of 300–550 K. The sensitivity obtained via the lifetime method was 7.3× lower than that measured via the FIR. Within the optimum temperature range of 300–460 K, the as-estimated sensor sensitivity was increased from 0.0013 to 0.028 K^−1^. With a further increase in temperatures, the sensitivity started to decline. A maximum relative sensitivity was estimated to be 2.22%K^−1^ at 460 K. The resolutions in both methods were below 1K in the above temperature range. The results indicated the suitability of SrZrO_3_:Eu^3+^ for the distinct high temperature sensing applications.

In daily human life, temperature is recognized as one of the most important measured physical quantities. An accurate and consistent measurement of temperature is essential for the devices related to chemistry, medicine, biology and metrology owing to the cooling and heating phenomena. In the modern world, temperature sensors are widely used, accounting for about 75–80% of the overall sensor market. The conventional temperature sensing measurement mainly depends on the ability of materials in response to heat. Direct contact of the instrument with the heated objects is the main requirement for making such thermometric measurements^1^,^2^,^3^.

Nowadays, non-contact specific thermometry with high resolution for sensing temperatures in nanoscale environments has emerged as a very dynamic field of research^4^. Among the non-invasive thermometric methods, luminescence thermometry has been established as the key alternative and an accurate technique with high detection sensitivity, spatial resolution and short acquisition times^5^,^6^,^7^. The temperature sensors using luminescent materials are based on the change in photoluminescence properties as a function of temperatures. The photoluminescence properties include the emission intensity, peak position, full width at half maxima of the emission spectrum and the characteristic lifetime of the excited state^8^,^9^.

Nikolic *et al.*^10^ suggested two ways to measure the thermometric behavior of luminescent materials accurately. The first method involves the measurement of the fluorescent intensity ratio (FIR) of the host emission to the emission related to the doped rare earth ions. In the second method, the time-dependence of the fluorescent intensity of a particular electronic transition is measured^11^,^12^. Based on the above two methods, luminescent thermometers can be fabricated by using various thermal probes, such as organic dyes, quantum dots (QDs) and rare earth ions. During the past decade, abundant research has been carried out based on organic dyes and QD thermometry. Nano-thermometers based on rare earth doped hosts with wide band gap have also been reported^1^,^2^,^3^,^4^,^5^,^6^,^7^,^8^,^9^,^10^,^11^,^12^. According to previous reports on luminescence thermometry, the temperature sensing via the measurements of emission lifetime requires more complex equipment. Regarding the luminescence thermometric applications, the FIR technique is found to be faster and simpler than the luminescence lifetime method^1^,^2^,^3^,^4^,^5^,^6^,^7^,^8^,^9^,^10^,^11^,^12^. Recently, increased interest is focused on developing thermometers using wide band gap hosts doped with suitable rare earth ions via the FIR method.

The performance of luminescent thermometers significantly depends on the photoluminescence efficiency of the corresponding luminescent materials^1^,^2^,^3^,^4^,^5^,^6^,^7^,^8^,^9^,^10^,^11^,^12^. Luminescent materials with different microstructures can be synthesized via different methods^13^. Recently, hollow spherical oxides have gathered enormous attention because of the stable self-supported structure, elevated crystallinity, and controlled nanosized inner space^14^,^15^,^16^. Unlike solid spheres, hollow spheres possess relatively low density, high surface area, high surface packing density, surface permeability and light-trapping effects. Therefore, hollow spherical structures are more advantageous in terms of energy transformation efficiency to achieve elevated luminescent efficiency and hence applicable to biolabels, drug delivery, and luminescent sensors^14^,^15^,^16^. The hollow structures are considered suitable hosts for many rare earth ions owing to outstanding thermal and environmental stability. Moreover, the low vibrational energy of hollow spherical structures is very suitable in minimizing the concentration quenching of the excited rare earth ions^17^. Adopting the hollow structure could also cut down the usage of raw materials as well as production cost in comparison with solid spheres^18^.

For various optoelectronic applications, alkaline-earth perovskite structured MZrO_3_ (M = Sr, Ba, Ca) are attractive candidates because of their high thermal as well as chemical stability and eco friendly nature^19^,^20^,^21^. The displacement of Zr or M atoms in disordered perovskite MZrO_3_ may induce some vacancy defects at the axial and planar oxygen sites of the [ZrO_6_] octahedral^19^. These vacancies act as luminescence centers owing to which the perovskite MZrO_3_ exhibits broad violet-blue emission. Zou *et al.*^20^ prepared SrZrO_3_ and BaZrO_3_ hollow micrometered particles which exhibited excellent adsorption capacities for efficient optoelectronic applications. With a wide band gap (~5.6 eV) and excellent physical and mechanical characteristics, rare earth doped SrZrO_3_ hollow spheres have potential applications in catalysis, chemical storage, ionic intercalation, light weight fillers, photonic crystals, and various optical devices^19^,^22^,^23^,^24^,^25^.

During the past few years, numerous researches have been carried out on the structural and photoluminescence properties of SrZrO_3_: Eu^3+^ nanocrystals synthesized via different routes^22^,^23^,^24^,^25^. Detailed literature review indicates that all the existing researches mainly focused on the synthesis, structural and optical characterizations of rare earth doped SrZrO_3_ sample. However, no research has attempted to probe into the luminescent thermometric characteristics of Eu^3+^ doped SrZrO_3_ hollow structures. It is worth mentioning that the sol-gel method is capable of producing hollow oxide particles^26^,^27^. The sol-gel method allows low temperature (~100 °C) processing, and has been proved to be very advantageous in controlling composition homogeneity and dispersion at molecular level. Variation of sol-gel synthesis conditions is very crucial to adjust the morphology of hollow microstructures. Furthermore, the sol-gel method provides better control over the size distribution of the produced powder in comparison with several other methods^26^,^27^.

In the present research, the hollow spherical structure of SrZrO_3_ was synthesized via the sol-gel method and the broad violet blue emission was observed upon UV excitations. Eu^3+^ ions were reasonably doped into the SrZrO_3_ host. Very little work has been done on luminescent thermometry based on trap emission and rare earth emission for the purpose of measuring temperature. To the best of the authors’ knowledge, SrZrO_3_: Eu^3+^ hollow spheres have not been used for the temperature sensing applications so far. In the present work, the trap emission of SrZrO_3_ host and the characteristic emission of Eu^3+^ ions were used to determine the FIR values in order to achieve luminescence thermometry. The sensor sensitivity and temperature resolution of Eu^3+^-doped SrZrO_3_ phosphor were calculated. Furthermore, the present research also aimed to compare the FIR technique with the fluorescent lifetime method, and the most suitable approach for the luminescence thermometry has been proposed.

## Results and Discussion

### Theoretical background

The thermometric behavior of a luminescent material includes the time-dependent intensity of a particular transition (lifetime decay curve) and the relative intensity (FIR)^11^,^12^. Electrons in the high excited states of materials generally relax toward low excited states or ground state via the combination of radiative and non-radiative transition processes. Therefore, the emission intensity (I) is proportional to the population density of the luminescent species in the excited states. The emission life-time depends on temperature according to the following equation^11^,^12^:


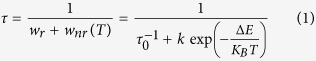


where *W*_*r*_ and *W*_*nr*_ are the probabilities of radiative and non-radiative decay processes, respectively, τ_0_ is the radiative lifetime at absolute zero, k is the pre-exponential factor, ΔE is the energy gap between the emitting level and the higher excited state, and k_B_ is the Boltzmann constant. Therefore, the temperature-dependence of decay time can be measured for the temperature sensing purposes.

The intensity of any optical transition is proportional to the total number of atoms (population) in a given excited state. At temperature T, the ratio of the fluorescence intensities of two transitions can be written as^11^,^12^:


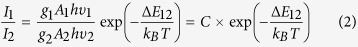


where g_1_ and g_2_ are the degeneracy of the respective states, *A* is the spontaneous emission rate, *h* is the Planck constant, *υ* is the frequency, *E* is the energy of the level, and C is a constant. Accordingly, the intensity ratio of the transitions can be used to measure the temperatures.

The above two methods are mutually related via the quantum efficiency of materials. The quantum efficiency of any emission is proportional to I and can be calculated using the following formula^5^,^10^:


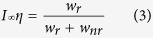


which implies that


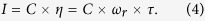


where C is a constant.

In the ideal case, the FIR is calculated as the ratio of the intensity of the temperature-dependent emission (I(T)) to that of the temperature-independent reference emission (I_R_)^5^,^10^:


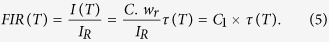


where T is the temperature. Equation (5) reveals that the FIR and time decay approaches are related to each other, and differ only in magnitude by a factor of C_1_ = C·w_r_/I_R_. However, measuring temperature by estimating the FIR requires two typical emission bands, one of which is used as a reference. Lifetime signals can be used with only one emission band to measure temperature. In the preset work, temperature sensing via the above two approaches has been described.

### Morphology evaluation

**Figure 1:**
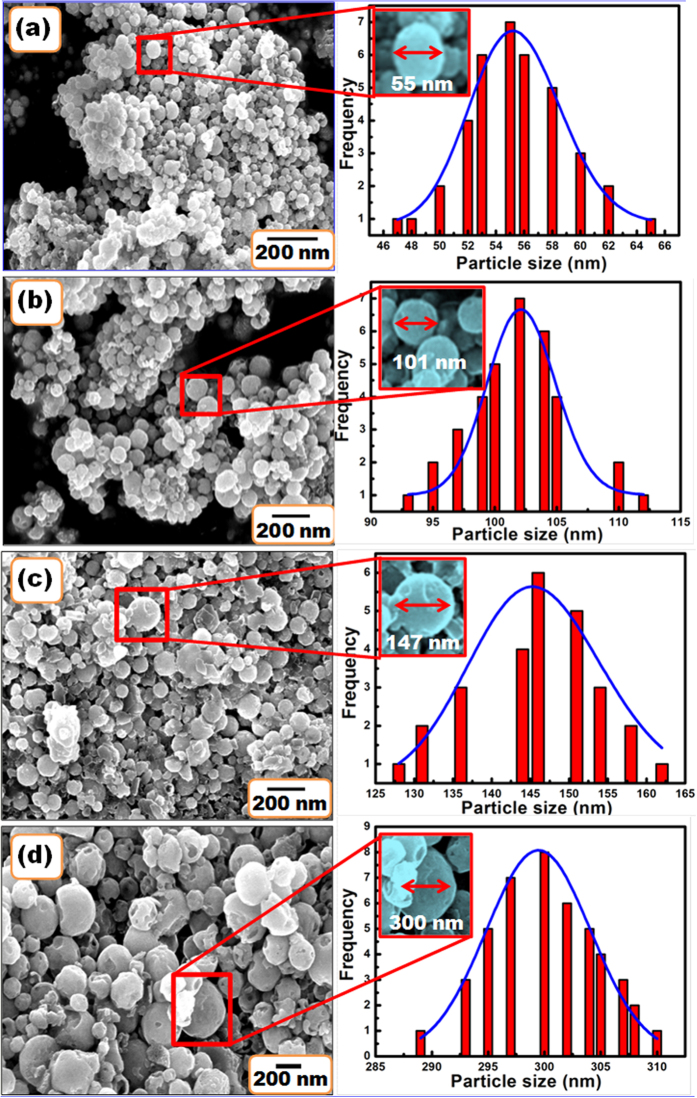
SEM images of SrZrO_3_:Eu^3+^ synthesized via the sol-gel method and post annealed at (**a**) 500 °C, (**b**) 700 °C, (**c**) 900 °C, and (**d**) 1100 °C. Inset in each figures show size distribution histograms.

Figure 1 shows the SEM images of SrZrO_3_:Eu^3+^ phosphor synthesized via the sol-gel route and annealed at various temperatures. As shown in Fig. 1(a), the synthesized particles annealed at 500 °C were spherical in morphology. The average particle size was estimated to be 50 nm. The particle size increased systematically with annealing temperature, as demonstrated in Fig. 1(b–d). The SEM images of the sample annealed above 700 °C revealed many destroyed spheres, indicating that the spheres are hollow in nature. The insets of each figure indicate the corresponding size distribution histograms. The diameters of the spherical shaped particles were found to obey the log normal behavior^28^:


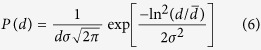


where *d* and *σ* are the average size and the size distribution of the particles, respectively. The average diameters of the sol-gel derived samples annealed at 500, 700, 900 and 1100 °C were estimated to be 55.39, 102.17, 145.77 and 300 nm, respectively. The corresponding size distributions were calculated to be (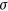
=) 0.58, 0.27, 0.55 and 0.16, respectively.

**Figure 2:**
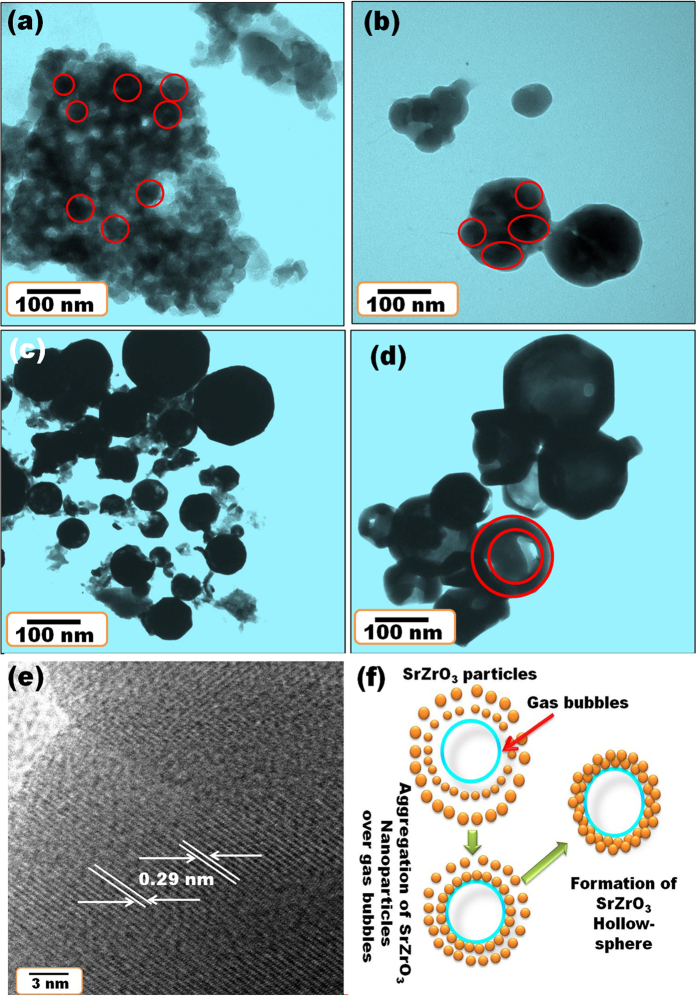
TEM images of SrZrO_3_:Eu^3+^ synthesized via the sol-gel method and post annealed at (**a**) 500 °C, (**b**) 700 °C, (**c**) 900 °C, and (**d**) 1100 °C. (**e**) HRTEM image of the SrZrO_3_:Eu^3+^ post annealed at 1100 °C. (**f** ) Schematic illustration of the formation of SrZrO_3_:Eu^3+^ hollow spheres.

The TEM images of SrZrO_3_:Eu^3+^ hollow spheres synthesized via the sol-gel route and annealed at various temperatures from 500 to 1100 °C are shown in Fig. 2(a−d). The results indicate an increase in size of the hollow spherical particles with annealing temperature. Figure 2(e) shows a high resolution lattice image of SrZrO_3_ particles post annealed at 1100 °C. It reveals the crystalline nature of the sample. The lattice pattern suggests the absence of any other impurity phase. The consecutive lattice fringes were arranged in order without any crystalline border. The spacing of the observed lattice fringes was calculated to be 0.29 nm, which was associated with the (002) lattice plane of the orthorhombic SrZrO_3_ (JCPDS 44–0161).

The morphological evolution suggests that SrZrO_3_:Eu^3+^ hollow microspheres were formed. During the synthesis process, zirconium cations were readily hydrolyzed in base solutions to form soluble Zr(OH)_5_^–^ anions. Meanwhile, strontium cations formed Sr(OH)^+^ species in the concentrated KOH solutions. The reaction between Zr(OH)_5_^–^ and Sr(OH)^+^ initiated the nucleation and growth of primary SrZrO_3_ particulates via the reaction Sr(OH)^+^ + Zr(OH)_5_^–^ = SrZrO_3_ + 3H_2_O. When the concentration of the KOH base solution was high, the solubility of the reactants was high and super-saturation of the reactant solutions was achieved. The concentrated base solutions favored the nucleation to form tiny primary particulates.

After heating at 500 °C, these primary particulates readily agglomerated into aggregated particles via reducing the high surface energy. By increasing the heating temperatures, the NO_2_ bubbles were formed. The produced NO_2_ plays a vital role in the growth of SrZrO_3_ hollow spheres. Small SrZrO_3_ nanocrystals may aggregate around the gas–liquid interface between NO_2_ and the solvent to reduce the interfacial energy. Finally, SrZrO_3_ hollow microspheres are formed. The scheme in Fig. 2(f) demonstrates the construction of SrZrO_3_ hollow spheres. Further increasing the heating temperature caused gradual disappearance of the core to generate hollow particles with slightly thickened shells. The sol–gel derived particles annealed at 1100 °C were used in the subsequent studies.

### Phase identification and structural refinement

**Figure 3:**
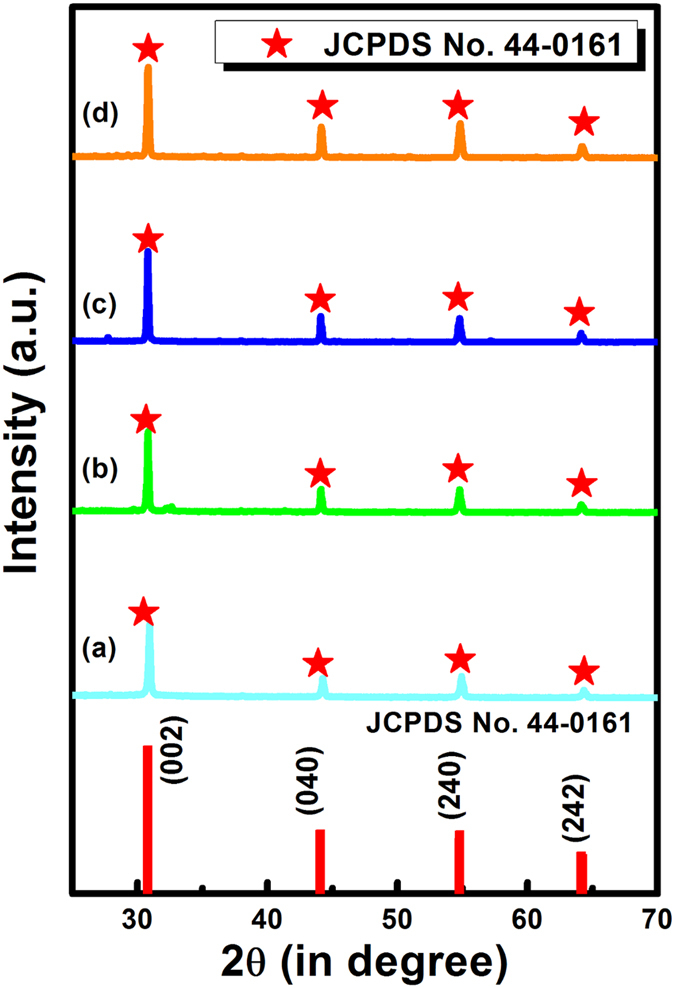
XRD patterns of SrZrO_3_:Eu^3+^ synthesized via the sol-gel method and post annealed at (**a**) 500 °C, (**b**) 700 °C, (**c**) 900 °C, and (**d**) 1100 °C.

Figure 3 shows the XRD patterns of sol-gel synthesized SrZrO3:Eu^3+^ followed by heating at various temperatures from 500 to1100 °C. All patterns matched well with the standard orthorhombic perovskite SrZrO_3_ phase (JCPDS 44–0161)^18^,^19^,^20^. As the annealing temperature was increased, the crystalline phase of SrZrO_3_:Eu^3+^ remained unchanged and no other phase was formed. The crystallinity of SrZrO_3_:Eu^3+^ increased significantly with elevated temperature, as demonstrated by the gradual sharpening of the diffraction peaks in Fig. 3.

**Figure 4:**
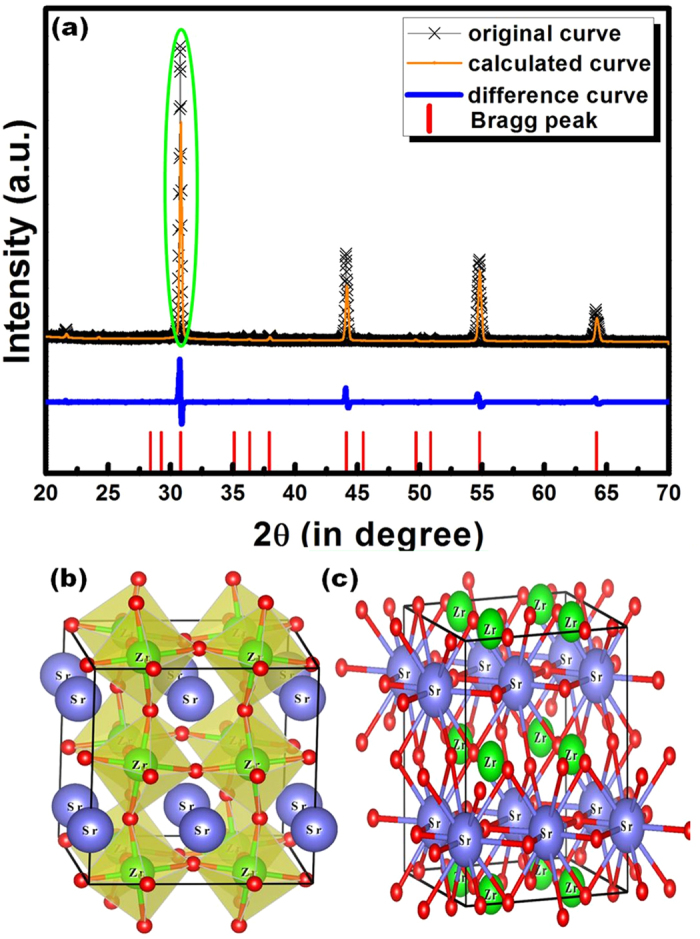
(**a**) Rietveld refinement pattern of SrZrO_3_:Eu^3+^ and (**b,c**) schematic illustration of the SrZrO_3_ structure.

Figure 4(a) shows the Rietveld refined XRD pattern of sol-gel synthesized SrZrO_3_:Eu^3+^ annealed at 1100 °C. The refinement was performed using *FullProf* software^29^. The “×” marks represent experimental diffraction data. The solid curves represent simulated diffraction data, the straight bars indicate the positions of simulated diffraction patterns, and the dotted lines represent the deviation between the simulated and experimental values. The refinement results reveal that the diffraction peaks are consistent with the orthorhombic perovskite structure with *Pnma* space group (JCPDS 44–0161). Table 1 presents the as calculated cell parameters.

The unit cell diagram of SrZrO_3_ is drawn with VESTA software^30^ using the data from Table 1 and is shown in Fig. 4 (b). Figure 4 (b) schematically depicts the coordination of Zr^4+^ ions in SrZrO_3_ host lattice and that of Sr^2+^ ions is shown in Fig. 4(c). According to the unit cell diagram, Sr^2+^ ions are located at the center of the lattice and coordinated with 12 oxygen ions. Zr^4+^ ions are located at the corners and coordinated with 6 oxygen atoms. Table 1 presents the as calculated cell parameters.

### Photoluminescence measurements

**Figure 5:**
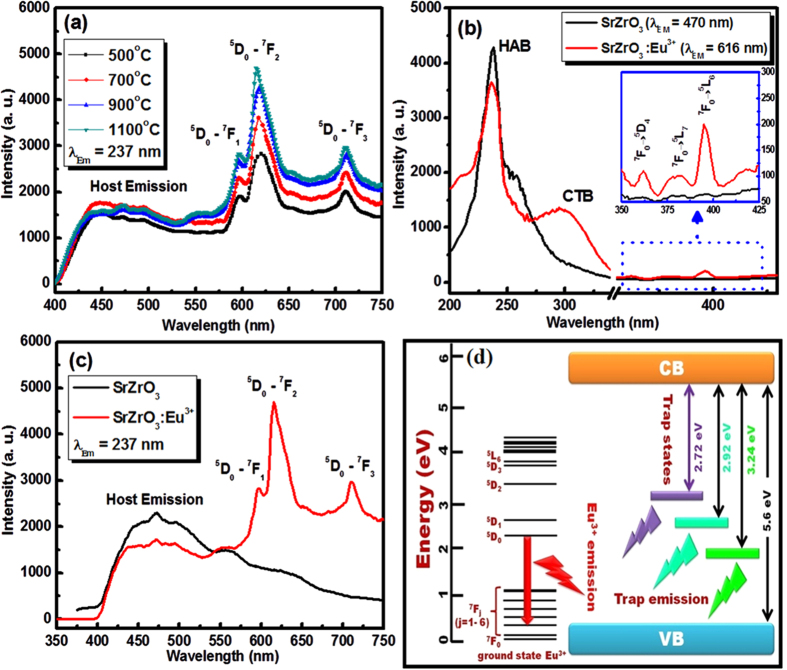
(**a**) Photoluminescence emission spectra of SrZrO_3_:Eu^3+^ synthesized via the sol-gel method and post annealed at various temperatures. Photoluminescence (**b**) excitation and (**c**) emission spectra of SrZrO_3_ and SrZrO_3_:Eu^3+^ synthesized via the sol-gel method and post annealed at 1100 °C. (**d**) Energy level scheme of trap emission and Eu^3+^ emission in SrZrO_3_:Eu^3+^.

Photoluminescence (PL) emission spectra of sol–gel derived SrZrO_3_:Eu^3+^ samples annealed at various temperatures were recorded using 237 nm UV excitation and the corresponding results are depicted in Fig. 5(a). The major and sharp emission lines at 595, 616 and 712 nm are attributed to ^5^D_0_ → ^7^F_J_ (J = 1, 2, 3) transitions of Eu^3+^ ions, respectively. The hypersensitive electric dipole transition observed at 616 nm (^5^D_0_ → ^7^F_2_) is found to be the strongest among all the emission lines^31^. The broad emission band located in the high-energy spectral region and peaked at 470 nm is associated with the trap emission of SrZrO_3_ host lattice^32^.

To examine the origin of the emission nature of SrZrO_3_:Eu^3+^, the excitation spectra of sol–gel derived SrZrO_3_ and SrZrO_3_:Eu^3+^ samples annealed at 1100 °C were monitored at the emission wavelengths of 470 and 616 nm, respectively (Fig. 5(b)). Under the emission wavelength of 616 nm, the excitation spectrum of SrZrO_3_:Eu^3+^ exhibited an intense peak at 237 nm (5.23 eV) belonging to the host absorption band (HAB) which is attributed to the charge transfer from oxygen ligands to central zirconium atom inside ZrO_3_^2−^ group^32^. The other broad peak centered at 295 nm was assigned to the charge transfer band (CTB) of europium ions (O^2−^ → Eu^3+^). Several narrow bands within the region of 360–470 nm generated from the *f-f* transitions within Eu^3+^:*4f *^6^ configurations were also observed. The excitation lines of Eu^3+^ ions are not prominent since the absorption intensity of the *f–f* transitions of Eu^3+^ ions in the longer wavelength region is very weak with respect to that of the ZrO_3_^2−^ groups. Therefore, the excitation of Eu^3+^ ions could be mostly due to the energy transfer (ET) from ZrO_3_^2−^ groups to Eu^3+^ ions.

Meanwhile, the excitation spectrum of SrZrO_3_, recorded under the emission wavelength of 470 nm, exhibited an intense HAB at 237 nm. As can be seen, the nature of HAB is almost identical in SrZrO_3_ and SrZrO_3_:Eu^3+^ samples, predominant at about 237 nm (5.23 eV), indicating that the UV irradiation energy can be efficiently absorbed by the present host. However, the excitation intensity of the HAB was lower in SrZrO_3_:Eu^3+^ with respect to SrZrO_3_, indicating that some of absorbed energy by the host might be transferred to the emission centers of Eu^3+^ ions. To confirm the ET between host and activators, the emission of SrZrO_3_ and SrZrO_3_:Eu^3+^ was also examined. As shown in Fig. 5(c), the ET process from host (oxygen-vacancy) to Eu^3+^ was confirmed, as the violet-blue emission was slightly reduced after Eu^3+^ doping into SrZrO_3_. Figure 5(d) presents a schematic energy level diagram for the trap emission and characteristic emission of Eu^3+^ ions from SrZrO_3_.

**Figure 6:**
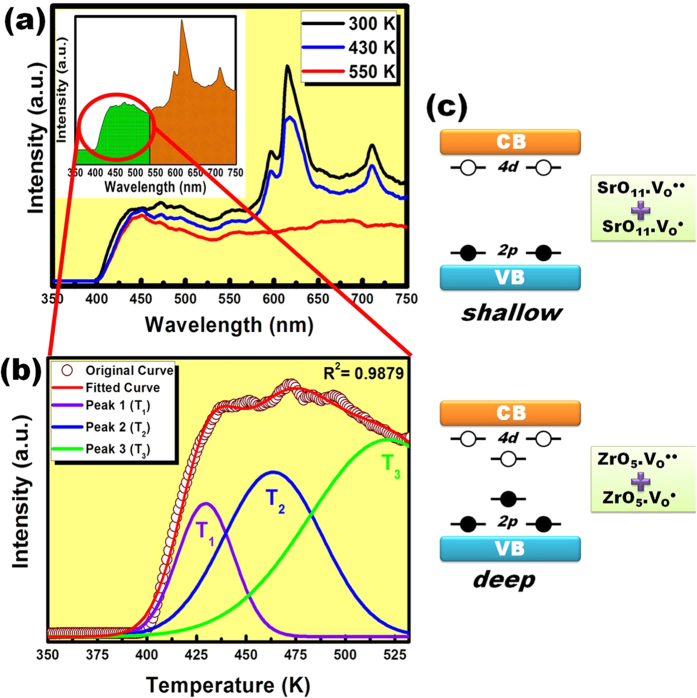
(**a**) Variation in photoluminescence emission spectra with temperature. Inset: Spectra showing two distinct spectral regions. (**b**) Deconvolution of the trap emission of SrZrO_3_ host. (**c**) Schematic illustration of deep and shallow level emission in SrZrO_3_ host.

The photoluminescence emission spectra of sol-gel derived SrZrO_3_:Eu^3+^ sample annealed at 1100 °C was obtained under excitation at 237 nm as a function of various temperatures (300–550 K). Figure 6(a) presents the PL emission spectra at 300, 430 and 550 K. All the spectra include two spectral regions, indicated as R1 and R2 in the inset in Fig. 6(a). Region R1 is the high-energy spectral region associated with the trap emission of the SrZrO_3_ host. The low-energy spectral region R2 is composed of well resolved emission peaks of Eu^3+^ ions.

The trap emission region (R1) of the spectrum of SrZrO_3_ host was deconvoluted using the Gaussian line broadening mechanism for luminescence processes. The deconvolution was performed to evaluate the exact positions of emission centers inside the band gap of SrZrO_3_ 32,33. Figure 6(b) presents three PL components that were obtained after peak fit deconvolutions. The three components are violet-blue at 430 nm (T_1_), blue-green at 463 nm (T_2_) and green at 525 nm (T_3_). According to Longo *et al.,* the violet and blue emission is attributable to shallow defects in the band gap, and the green emission is attributable to defects that are deep within the band-gap and arise from the oxygen vacancies^34^. The blue-green emission is associated with surface defects^32^,^33^,^34^.

Longo *et al.*^34^ and Guo *et al.*^35^ explained the effect of oxygen vacancies (V_o_) via estimating the density of states associated with the emission of SrZrO_3_. The refinement studies and the proposed coordination of Zr^4+^ and Sr^2+^ ions in SrZrO_3_ host lattice (Fig. 4) revealed that Zr^4+^ ions are coordinated with 6 oxygen atoms and Sr^2+^ ions are coordinated with 12 oxygen ions. Accordingly, [ZrO_5_.V_o_^..^] and [ZrO_5_.V_o_^.^] complex clusters were formed with the help of oxygen vacancies and therefore increased the disorder in the lattice^34^. Such complex defects are deep within the band-gap, leading to green–yellow–red photoluminescence emission. In contrast, [SrO_11_.V_o_^..^] and [SrO_11_.V_o_^.^] complex clusters are associated with shallow defects in the band–gap and lead to violet–blue light emission. Figure 6(c) presents a schematic diagram of the deep and shallow defects^34^.

### Measurement of FIR and lifetime data at various temperatures

The emission associated with doping ions declines rapidly with temperature due to the enhanced non-radiative relaxation. However, the changes in trap emission from SrZrO_3_ host with a rise in temperature are minor. Therefore, the trap emission from SrZrO_3_ host can provide the reference intensity I_R_ for the FIR measurement. The intensity I_R_ was measured as the area under the emission curve associated with the trap transitions that is equivalent to the spectral area in the range of 400–535 nm. The intensity of the temperature-dependent emission I(T) was obtained from the measured spectral area in the range of 540–675 nm. The ratio of I(T) to IR yielded the value of FIR.

**Figure 7:**
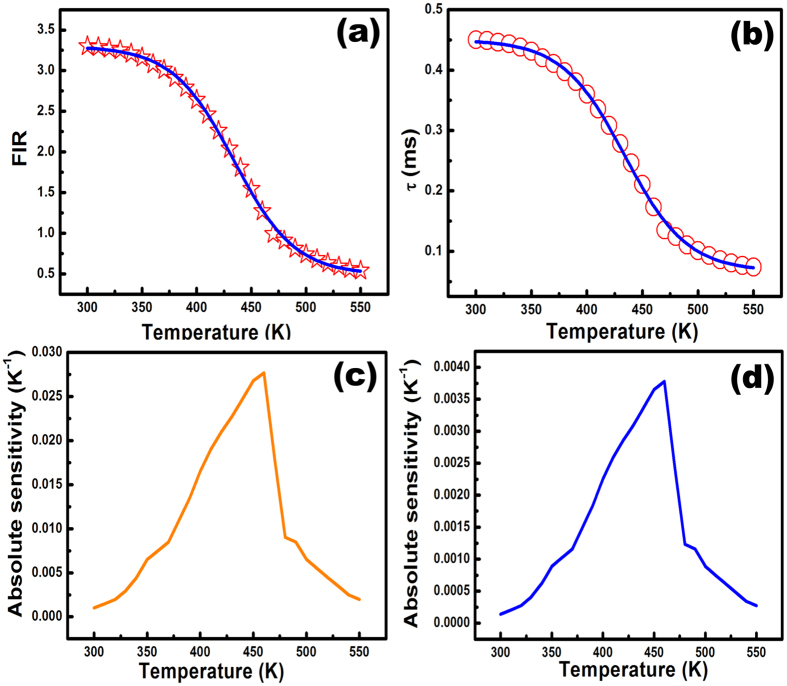
Variation in (**a**) FIR value and (**b**) lifetime value of ^5^D_0_ state of Eu^3+^ ions in SrZrO_3_ host with temperature. The blue solid line indicates the dependence with Boltzmaan distribution. Variation in absolute sensor sensitivity with temperatures evaluated via measuring (**c**) FIR value and (**d**) lifetime value of ^5^D_0_ state of Eu^3+^ ions in SrZrO_3_ host.

Figure 7(a) shows the temperature-dependence of the FIR data. Figure 7(b) plots the temperature-dependence of the lifetime data obtained via monitoring the 616 nm emission of Eu^3+^ ions at various temperatures. Both sets of data exhibited the same behavior in the studied temperature range and consistent with Eq. (4). Therefore, the temperature can be measured by dividing the whole spectrum into two regions rather than by measuring two particular transitions, which substantially simplifying the thermometric measurement in the present case. Figure 7 clearly reveals that the FIR and lifetime values decline by approximately an order of magnitude as the temperature varies in the range of 300–550 K. Such variation is one of the main requirements of an efficient temperature sensor. Therefore, the obtained materials herein can be effectively used for sensing temperature.

### Absolute and relative sensitivity and the temperature resolution

The performance of a temperature sensor typically depends on the figure of merit of the sensing behavior. The figure of merit includes various parameters such as absolute sensitivity (S_a_), relative sensitivity (S_r_) and resolution. The absolute sensitivity is defined as the variation of the FIR or lifetime (in the two approaches) with temperature, and is given by^36^,^37^:


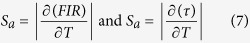


According to the present analysis, the as-calculated absolute sensor sensitivity was increased from 0.0013 K^−1^ to 0.028 K^−1^ as the temperature increased from 300 K to 460 K. With a further rise in the temperature, the sensitivity declined. Figure 7(c) presents the variation in absolute sensitivity with temperature for FIR measurement. Figure 7(d) plots the similar variation in absolute sensitivity with temperature for lifetime measurement. However, the sensitivity was approximately 7.3 times lower than that measured via the FIR.

The relative sensor sensitivity is the absolute sensor sensitivity normalized with respect to the measured value. The relative sensitivity can be calculated as follows^35^,^36^:


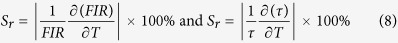


**Figure 8:**
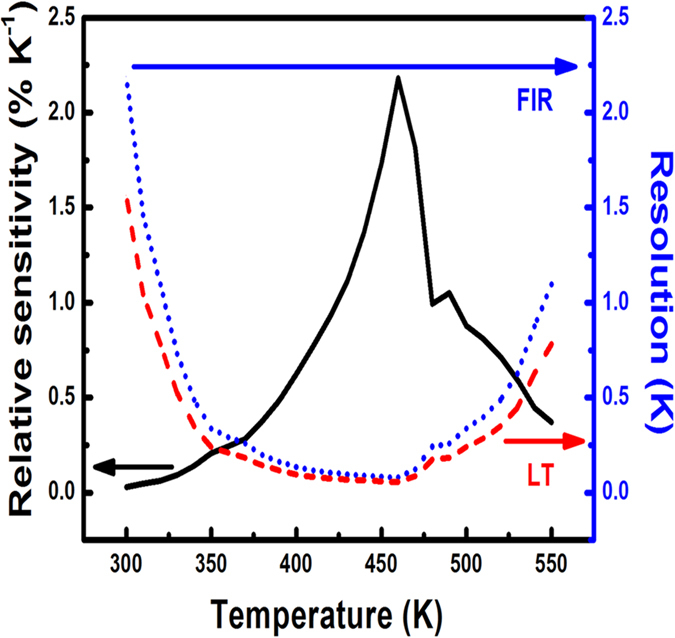
Variation in relative sensor sensitivity and resolution with temperature. The black solid line indicates relative sensor sensitivity, the blue dashed line implies to the resolution calculated via FIR approach and the red dashed line implies to the resolution calculated via lifetime approach.

Since the FIR and lifetime values depend equally on temperature, the relative sensor sensitivities calculated using both methods are represented via the black line in Fig. 8. It is revealed that the relative sensor sensitivity increases with temperature up to 460 K and then decreases. However, the sensitivity is reasonably higher than the reported value for a wide temperature range from the room temperature upwards (Table 2). The above finding reveals the suitability of Eu^3+^-doped SrZrO_3_ hollow spheres for sensing temperature in various electronic devices. The maximum relative sensor sensitivity was 2.22% K^−1 ^at 460 K. Table 2 compares recently developed Ln^3+^ phosphor-based inorganic nano-thermometers in terms of relative sensor sensitivity and the temperature range. As shown in Table 2, the present samples herein yield the highest sensor sensitivity.

The temperature resolution is also an important characteristic of any temperature sensing device and can be defined as the minimal detectable change in signal^49^,^50^,^51^,^52^. The standard deviation of the residuals in the fit of the FIR/lifetime data as a function of temperature and the absolute sensitivity were adopted to estimate the resolution using the method described by Brites *et al.*^5^. Figure 8 presents the estimated resolutions of FIR and lifetime temperature sensing. The two curves are similar. However, the lifetime measurements provide a higher resolution than the FIR. The resolutions in both cases are lower than 1K over a wide temperature range of 310 to 540 K. In both methods, the maximal resolution was obtained at 460 K. The estimated resolution was 0.13 K for the lifetime measurements and 0.16 K for the FIR. The comparison of SrZrO_3_:Eu^3+^ system with the recently reported phosphors (Table 2) in terms of sensitivity and resolution reveals the suitability of the material for temperature sensing applications. A brief comparison of the hollow and solid structured SrZrO3:Eu^3+^ spheres in respect of the PL performance and FIR sensitivity to temperature has also been provided as the supplementary information. The maximum relative sensitivity of the solid spherical particles was estimated to be around 0.75% K^−1^ at 410 K. The results indicate that the sensitivity and resolution of hollow spheres are better than those of solid sphere. Hence, the hollow spherical SrZrO_3_:Eu^3+^ are more suitable for the thermometric applications than its solid form.

## Conclusions

In conclusion, Eu^3+^-doped SrZrO_3_ hollow spheres were successfully obtained via the sol-gel synthesis method followed by heating at various temperatures ranging from 500 to 1100 °C. Structural characterizations revealed the formation of orthorhombic perovskite phase. The crystallinity and photoluminescence intensity increased with heating temperature owing to the reduction of surface defects. The photoluminescence emission spectrum of SrZrO_3_: Eu^3+^ hollow spheres exhibited two spectral zones. The spectral zone at the low wavelength region was attributed to trap emissions from the SrZrO_3_ host. The sharp red emission at the high wavelength region was attributed to the emission of Eu^3+^ ions. The fluorescent intensity ratio of the emissions of Eu^3+^ ions to the SrZrO_3_ trap emissions depended strongly on temperature, and was therefore studied for sensing temperature. The maximum sensitivity was estimated to be 2.22%K^−1^ at 460 K with the resolution of 1 K, indicating suitability of the material for temperature sensing applications.

## Materials and Methods

Perovskite SrZrO_3_: 2mol%Eu^3+^ samples were synthesized via the sol-gel method using strontium nitrate (Sr(NO_3_)_2_), zirconyl nitrate hydrate (H_2_N_2_O_8_Zr), europium oxide (Eu_2_O_3_) and nitric acid (HNO_3_) as the starting raw materials. All chemicals were of analytical grade and supplied by Sigma-Aldrich. Required proportions of Eu_2_O_3_ were dissolved in the appropriate amount of HNO_3_ solution and heated on a hot plate to yield nitrates. 20 ml of deionized water was added to the residual nitrates, and appropriate proportions of H_2_N_2_O_8_Zr and Sr(NO_3_)_2_ were added to the solution. 20 ml of KOH solution with a concentration of 15 mol/l was added to the main solution. Then the final solution was stirred at 100 °C until the formation of gel. Finally, the mixtures were transferred into alumina crucibles and heated at various temperatures (from 500–1100 °C) for 12 h. In order to compare the PL and thermometric performance between the hollow and solid spherical morphologies of SrZrO_3_:Eu^3+^ particles, SrZrO_3_:Eu^3+^ solid nanospheres were also prepared via the sol-gel method followed by annealing at 1100 °C for 12 h. For the synthesis of solid nanospheres, the concentration of the KOH solution was adjusted to ~5 mol/l.

The crystalline structures of the prepared powders were identified via X-Ray diffraction (XRD; Philips X’pert/MPD, Amsterdam, the Netherlands) using *CuKα* radiation at room temperature. The microstructures and particle sizes were examined using scanning electron microscopy (SEM, Hitachi S-800) and high resolution transmission electron microscopy (TEM; JEM-3010, JEOL, Tokyo, Japan). The photoluminescence spectra of the synthesized phosphors were recorded using a fluorescence spectrophotometer (Hitachi F–4500, Tokyo, Japan) with a xenon lamp that was operated at 150 W as an excitation source. The obtained samples were heated on a hot plate combined with the temperature controller. The temperature was measured using a thermocouple that was in contact with the sample. Photoluminescence measurements were carried out within the temperature range of 300–550 K.

## Additional Information

**How to cite this article**: Das, S. *et al.* Structural evaluations and temperature dependent photoluminescence characterizations of Eu^3+^-activated SrZrO_3_ hollow spheres for luminescence thermometry applications. *Sci. Rep.* **6**, 25787; doi: 10.1038/srep25787 (2016).

## Supplementary Material

Supplementary Information

## Figures and Tables

**Table 1:** Refinement parameters of SrZrO_3_: 2 mol% Eu^3+^.

Sample	SrZrO_3_: 2%Eu^3+^
Crystal Syntem	Orthorhombic
Space Group	*Pnma*
a	8.208 Å
b	8.208 Å
c	8.196 Å
volume	552.175 (Å)^3^
α=β=γ	90°
R_wp_	9.67
R_p_	8.16
R_e_	6.4

**Table 2:** Comparative analysis of relative sensitivity (S_r_), peak temperature (T_m_) and the corresponding temperature range (ΔT) of luminescence sensor materials.

Serial No.	Phosphor	S_r_ at T_m_(in %K^−1^)	ΔT (T_m_) (in K)	Ref.
1.	CaF_2_: Er^3+^/Yb^3+^	2.30	293–318 (318)	38
2.	CaF_2_: Tm^3+^/Yb^3+^	0.20	293–318 (315)	38
3.	NaYF_4_: Er^3+^/Yb^3+^	1.00	298–318 (298)	39
4.	Gd_2_O_3_: Er^3+^/Yb^3+^	0.20	295–1000 (600)	40
5.	Fluoride glass: Er^3+^/Yb^3+^	1.10	333–375 (342)	41
6.	ZnO: Er^3+^/Yb^3+^	0.60	273–473 (273)	42
7.	GdVO_4_:Er^3+^/Yb^3+^	1.11	307–473 (307)	43
8.	YAG: Ce^3+^	0.20	315–350 (350)	44
9.	Y_2_O_3_: Eu^3+^	2.60	473–973 (973)	45
10.	SrY_2_O_4_:Eu^3+^	5.53	293–473 (473)	46
11.	BaY_2_ZnO_5_:Eu^3+^	2.20	330–510 (490)	47
12.	TiO_2_: Eu^3+^	2.43	307–533 (533)	5
13.	Gd_2_Ti_2_O_7_:Dy^3+^	1.68	293–443 (293)	48
14.	SrZrO_3_: Eu^3+^	2.22	300–550 (460)	Present work
