# Supplementary material for: Structural evaluations and temperature dependent photoluminescence characterizations of Eu3+-activated SrZrO3 hollow spheres for luminescence thermometry applications
Source: Sci Rep. 2016 May 18;6:25787. doi: 10.1038/srep25787 (PMC4870567; doi:10.1038/srep25787)
Supplement: Supplementary Information [file srep25787-s1.pdf]

Supplementary information

Structural evaluations and temperature dependent photoluminescence characterizations of Eu^{3+}-activated $SrZrO_3$ hollow spheres for luminescence thermometry applications

Subrata Das,[#] Sudipta Som,[#] Che-Yuan Yang, Sudam Chavhan, Chung-Hsin Lu*

Department of Chemical Engineering, National Taiwan University, Taipei, Taiwan, ROC Tel: +886-2-23651428, Fax: +886-2-23623040

Corresponding author: chlu@ntu.edu.tw

[#]These authors contributed equally

In order to compare the photoluminescence (PL) and luminescence thermometric performance between the hollow and solid spherical morphologies of $SrZrO_3:Eu^{3+}$particles, $SrZrO_3:Eu^{3+}$solid spherical particles were also prepared via the sol-gel method as mentioned in the Materials and Methods section of the main text. The X-ray diffraction (XRD) pattern of $SrZrO_3:Eu^{3+}$ solid spherical particles was matched well with that of standard orthorhombic perovskite $SrZrO_3$ phase (JCPDS 44−0161), as shown in Fig. S1 (a). The Scanning electron microscopic (SEM) image of $SrZrO_3:Eu^{3+}$ solid spherical particles confirmed the spherical morphology, as shown in supplementary Fig. S1 (b). The average particle size was estimated to be ~65 nm and a narrow size distribution was observed (Fig. S1 (c)).

Figure S2 (a) shows the comparative photoluminescence emission spectra of hollow and solid spherical $SrZrO_3:Eu^{3+}$ keeping excitation wavelength fixed at 237 nm. As illustrated in supplementary Fig. S2 (a), the ratio of the integral area for Eu^{3+} emission to the host emission of $SrZrO_3:Eu^{3+}$ solid spheres is greater than that of the hollow spherical $SrZrO_3:Eu^{3+}$ particles. The variation in fluorescent intensity ratio (FIR), relative sensitivity and sensor resolution of $SrZrO_3:Eu^{3+}$ solid spheres were recorded in the temperature range of 300–550 K, as depicted in Fig. S2 (b)–(d), respectively. These figures indicate that the sensitivity and resolution

of hollow spheres are better than those of the solid spheres. The maximum relative sensitivity was estimated to be around 0.75% K^{-1} at 410 K in the case of $SrZrO_3:Eu^{3+}$ solid spherical particles. However, the maximum sensitivity of hollow spherical $SrZrO_3:Eu^{3+}$ particles was estimated to be 2.22%K^{-1} at 460 K with the resolution of 1 K. Unlike solid spheres, hollow spheres possess relatively low density, high surface area, high surface packing density, surface permeability and light-trapping effects. These characteristics of hollow spherical structures are more advantageous for achieving high luminescence sensor sensitivity [14–16].

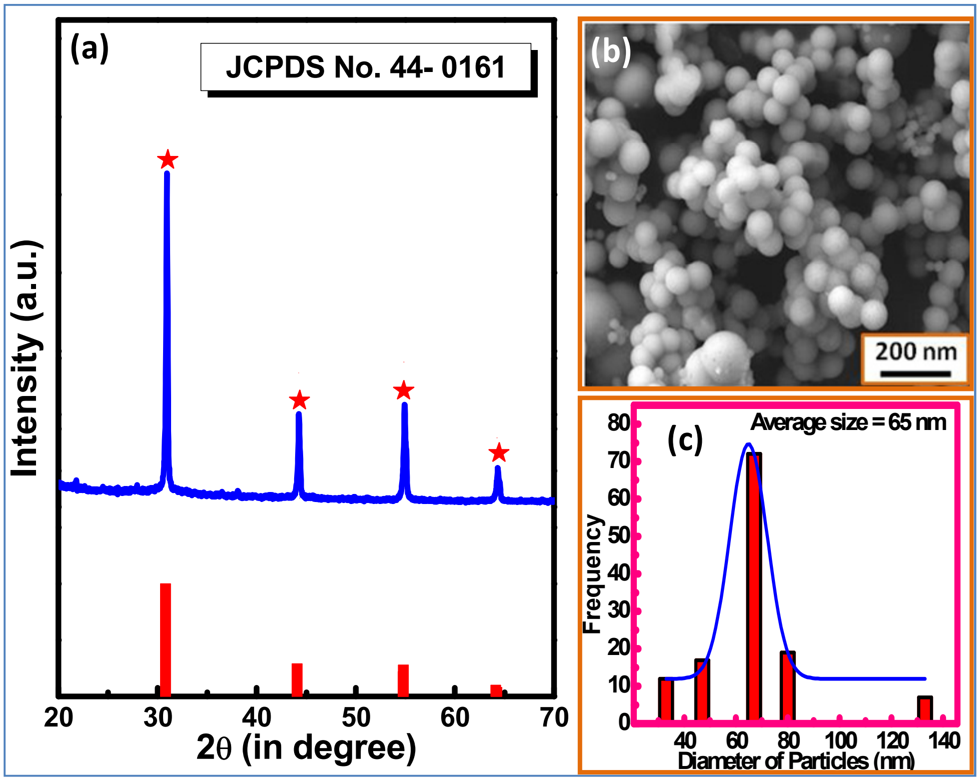

Supplementary Figure S1: (a) XRD pattern and **(b)** SEM images of $SrZrO_3:Eu^{3+}$ solid spheres. **(c)** Particle size distribution of $SrZrO_3:Eu^{3+}$ solid spheres derived from the corresponding SEM micrograph.

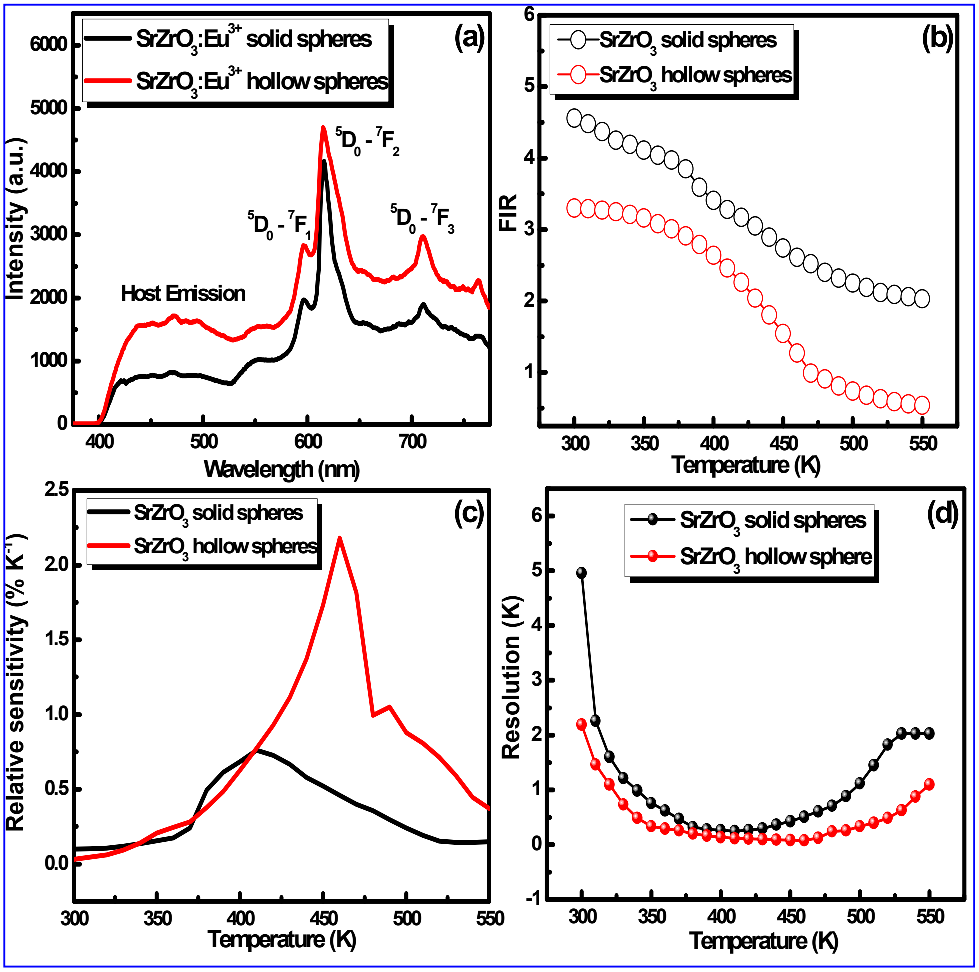

Supplementary Figure S2: (a) Photoluminescence emission spectra (λ_{Ex} = 237 nm) of $SrZrO_3:Eu^{3+}$ solid spheres. Variation in **(b)** FIR, **(c)** sensitivity and **(d)** resolutions of $SrZrO_3:Eu^{3+}$ solid spheres.
